# Population Structure and Genetic Diversity of the 175 Soybean Breeding Lines and Varieties Cultivated in West Siberia and Other Regions of Russia

**DOI:** 10.3390/plants12193490

**Published:** 2023-10-06

**Authors:** Nadezhda A. Potapova, Alexander S. Zlobin, Roman N. Perfil’ev, Gennady V. Vasiliev, Elena A. Salina, Yakov A. Tsepilov

**Affiliations:** 1 Kurchatov Genomic Center, Institute of Cytology and Genetics, Siberian Branch of the Russian Academy of Sciences, 630090 Novosibirsk, Russia; 2 Institute for Information Transmission Problems (Kharkevich Institute) of the Russian Academy of Sciences, 127051 Moscow, Russia; 3 Federal Research Center, Institute of Cytology and Genetics SB RAS, 630090 Novosibirsk, Russia

**Keywords:** soybean, *Glycine max*, phenotyping, genotyping, population structure, molecular diversity, Russia, plant breeding

## Abstract

Soybean is a leguminous plant cultivated in many countries and is considered important in the food industry due to the high levels of oil and protein content in the beans. The high demand for soybeans and its products in the industry requires the expansion of cultivation areas. Despite climatic restrictions, West Siberia is gradually expanding its area of soybean cultivation. In this study, we present the first analysis of the population structure and genetic diversity of the 175 soybean *Glycine max* breeding lines and varieties cultivated in West Siberia (103 accessions) and other regions of Russia (72 accessions), and we compare them with the cultivated soybean varieties from other geographical locations. Principal component analysis revealed several genetic clusters with different levels of genetic heterogeneity. Studied accessions are genetically similar to varieties from China, Japan, and the USA and are genetically distant to varieties from South Korea. Admixture analysis revealed four ancestry groups based on genetic ancestry and geographical origin, which are consistent with the regions of cultivation and origin of accessions and correspond to the principal component analysis result. Population statistics, including nucleotide diversity, Tajima’s D, and linkage disequilibrium, are comparatively similar to those observed for studied accessions of a different origin. This study provides essential population and genetic information about the unique collection of breeding lines and varieties cultivated in West Siberia and other Russian regions to foster further evolutionary, genome-wide associations and functional breeding studies.

## 1. Introduction

Soybean (*Glycine max*) is an important species in the food industry due to the high levels of oil and protein concentration in the beans. It is grown almost worldwide with large soybean production quantities related mainly to North and South America (e.g., the USA, Canada, Brazil, Argentina, and Paraguay), China, India, and East European countries [1]. Soybean has a broad application in the food industry as a source of protein, oil, and other nutrients for humans and livestock, and it is also used for man-made industrial products, i.e., plastics, clothes, fuels, etc. [2,3,4]. Varieties of this species have already been studied [5,6,7,8], while comparative population information about the Russian cultivars has remained unexplored. Russian cultivars widely vary in origin and genetic diversity due to geographical and climatic conditions in different parts of the country from the Far East, Western Siberia, and Ural to the Volga, Southern, and Central (Chernozem) regions [9].

Moreover, an active expansion of soybean cultivation areas recently occurred, including Western Siberia regions; therefore, it is of interest to compare samples created for this region with previously studied samples from areas of traditional soybean cultivation in various regions of the world.

Studies of the population and genetics of soybean accessions are necessary and important as they provide opportunities to uncover genetic relationships between varieties from different countries and regions to discover varieties and, consequently, loci and polymorphisms that are potentially important in selection to reveal genetic patterns relating to certain varieties, e.g., from certain countries and geographical locations, etc.

There are many examples of studies on soybean population genetics [5,10,11,12,13,14,15,16,17], whole-genome SNP arrays [7,8,9,10], or whole genomic data [11]. These studies uncover comprehensive information about the origin, relatedness between different varieties, genetic differentiation, diversity, and evolutionary forces acting on their genomes. However, different types of genotyping technologies and datasets (i.e., certain genes, genomes, or SNP arrays, etc.) were used in these studies, raising a problem for comparing measured population characteristics such as genetic diversity (π and θ) [18,19], linkage disequilibrium (LD) [20], Tajima’s D [21], etc. Nowadays, information obtained from a set of genes is not enough for studies of population genetics, and there is a possibility of using whole-genome sequencing or SNP arrays. An SNP array is an informative and affordable method; for instance, the Illumina Infinium SoySNP50K iSelect BeadChip [22] was suggested in 2013 and has been used in many studies, confirming its usability [23,24,25]. Hence, as this SNP array has previously been used in other soybean studies, it gives an opportunity to compare results from the same platform and obtain clear differences and similarities.

In this study, we present the first population structure and genetic diversity analysis—including the principal component analysis (PCA), nucleotide diversity π and θ, and linkage disequilibrium of the 175 soybean *Glycine max* accessions—cultivated in West Siberia and other Russian regions, compare them with the cultivated soybean varieties from other countries (China, USA, etc.), and discuss genetic similarities and differences between them.

## 2. Material and Methods

### 2.1. Russian Soybean Varieties

The 185 soybean *Glycine max* accessions including 96 breeding lines from West Siberia and 88 Russian and European cultivars, with 1 wild accession, were stored and multiplied in West Siberia by the Siberian Federal Scientific Center of Agro-BioTechnologies of the Russian Academy of Sciences (SFSC RAS, Novosibirsk, Russia) (Table S1). Eleven soybean cultivars were kindly provided by the Federal Scientific Center of Legumes and Groat Crops (FSC LGC, Orel, Russia). The soybean accessions are described in Perfil’ev and the co-authors [26]. Most of the accessions in the collection were collected by breeders from the Siberian Federal Research Center of Agro-BioTechnologies of the Russian Academy of Sciences (SFSCA RAS) (Novosibirsk) and were used as the main plant material for the selection of new cultivars. Novosibirsk is located at 55 degrees north latitude, which is quite an atypical condition for such a thermophilic and photoperiod-sensitive crop as soybeans. Thus, the study of this population may be interesting from the point of view of the biology of soybean adaptation to atypical conditions.

### 2.2. Genotyping

Genomic DNA was extracted using the CTAB method from 3- to 4-day-old seedlings grown in Petri dishes according to the method described earlier by [27]. The genotyping of 179 soybean accessions was performed for 52,041 SNPs using SoySNP50K iSelect BeadChip array [22] in the Genomic Centre of ICG SB RAS. The raw data were analyzed using Genome Studio v2 (Illumina Inc., San Diego, CA, USA) and converted to Plink format. As a reference genome, we used Wm82.a1, obtained from the SoyBase database (https://soybase.org/snps/, accessed on 25 September 2023). This version of the reference genome was chosen because the BeadChip array was developed for this version.

### 2.3. Soybean Dataset from SoyBase

An additional dataset of genotyped soybean accessions was retrieved from [28], available on SoyBase (https://soybase.org/snps/, accessed on 25 September 2023). It includes 20,087 *G. max* and *G. soja* accessions genotyped with 42,509 SNPs. Only *G. max*-related datasets were used in our study.

### 2.4. Data Quality Control

**Figure 1:**
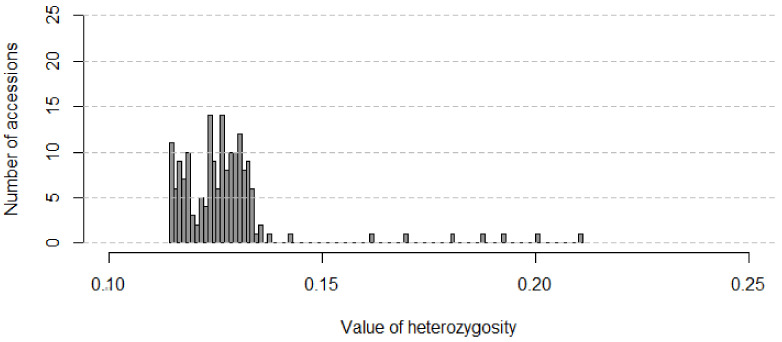
Histogram of heterozygosity per sample in 175 soybean accessions cultivated in West Siberia and other regions of Russia.

The dataset with 179 soybean accessions and 52,041 SNPs was filtered with the following criteria: --geno 0.05 --maf 0.05 --mind 0.05 using Plink (v1.90b6.26 [29]). Next, the samples with high heterozygosity (>0.3) were removed from the analysis using Plink (Figure 1). A wild soybean accession was also removed at this stage. The same filtration was performed separately for the additional dataset with *G. max* varieties.

The merging procedure between our dataset and the additional dataset was performed using Plink. The merged dataset was subjected to quality control with the same parameters as mentioned above.

### 2.5. Population Genetic Analysis

We performed PCA for 175 studied accessions. In addition, we performed PCA for a joint dataset of 175 accessions and the SoyBase dataset. We also performed PCA for a joint dataset of 175 accessions and varieties from the SoyBase dataset marked with the origin “Russia”. Principal component analysis, kinship analysis, and estimation of heterozygosity were performed using Plink. For admixture analysis, we used Admixture (v.1.3.0 [30]). At first, admixture analysis was performed with different K values (from 1 to 15) to obtain cross-validation error for each K and, consequently, to get the most probable number of clusters with the minimal cross-validation error, which was 4. Then, we ran the analysis using this K value and under default parameters.

Linkage disequilibrium values were obtained from the TASSEL tool (v.5.2.84 [31]) with the default parameters, and the method described in [32] was used for visualization. π, θ, and Tajima’s D values were also calculated in TASSEL. For the visualization of all the results, we used R scripts (version 2022.07.0, build 548).

## 3. Results

As a result of quality control, 29,724 SNPs and 175 soybean accessions passed through the filtration. For the other dataset, 16,986 samples and 26,741 SNPs passed quality control.

In general, SNPs in the analyzed dataset of 175 soybean accessions were distributed equally between chromosomes, considering chromosome length, and there were just a few SNPs located on the “Unknown” chromosome (Supplementary Figure S1).

Allele frequency distribution (Supplementary Figure S2) had two distinct peaks, which might be explained by the origin of this data from the SNP array and unequal number of SNPs mapped to different chromosomes.

The distribution of heterozygosity values (Figure 1) shows that studied soybean accessions have low levels of heterozygosity with a mean of 0.127 (standard deviation 0.014). This is primarily due to the peculiarity of the studied Russian collection, which consists of breeding lines and varieties maturing under the climatic conditions of Western Siberia [26].

**Figure 2:**
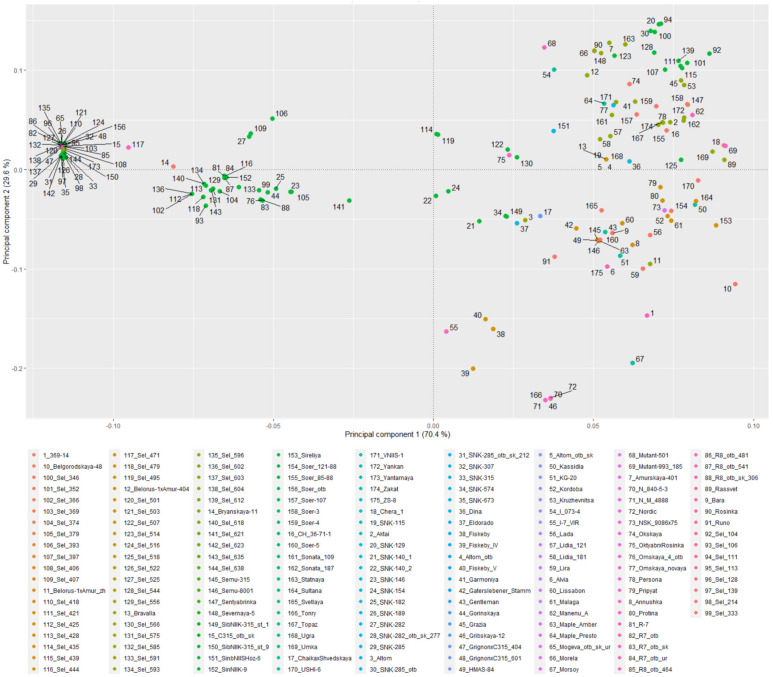
Principal component analysis for 175 studied accessions cultivated in West Siberia and other regions of Russia.

**Figure 3:**
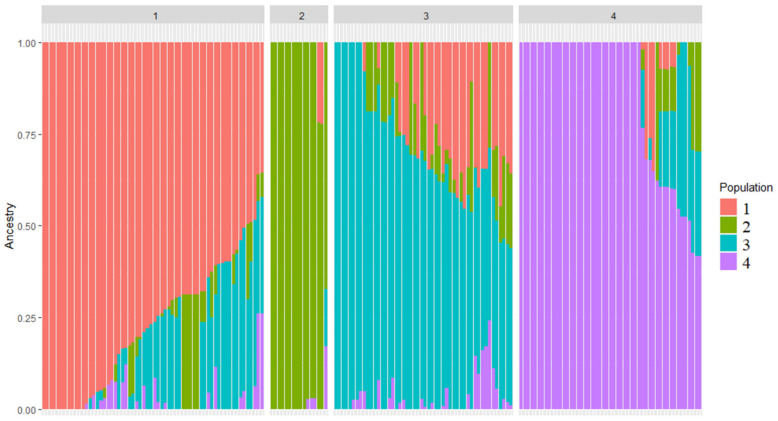
Admixture analysis for 175 analyzed soybean accessions. Colors represent genetic ancestries (red, green, cyan, and violet), and numbers represent clusters of origin.

The population genetic structure of studied soybean accessions is described using the PCA (Figure 2). There are two clusters on the left, which show high genetic similarity and consist of lines from breeding plots of West Siberia (from 75% to 80% of lines) (Tables S1 and S2). On the right side of the PCA, there are samples with much less genetic similarity. This reflection of their genetic heterogeneity is due to the fact that two clusters stand out in this group (clusters 3 and 4) according to admixture analysis (Figure 3). Cluster 3 consists of 28% breeding lines and 30% of accessions from the Far East. Cluster 4 consists of the accessions of European (e.g., France and Austria) origin more than other clusters. The plot describing percentage of the genetic variation explained by the first 10 principal components is presented in Supplementary Figure S3.

Kinship analysis supported the results of PCA, showing that two clusters on the left are very genetically similar within themselves—there were 62 pairs of accessions with an IBS (identity-by-state) coefficient higher than 75%.

Admixture analysis revealed four clusters of origin for studied soybean accessions (Figure 3, Supplementary Figure S4).

Linkage disequilibrium analysis (Figure 4) showed that LD half-life is about 1.2 Mb, and mean LD value is 0.33 Mb. The results for each chromosome are presented in Supplementary Figures S5–S24. The nucleotide diversity π in the analyzed dataset was 0.33, θ was 0.17, and Tajima’s D was equal to 2.94.

**Figure 5:**
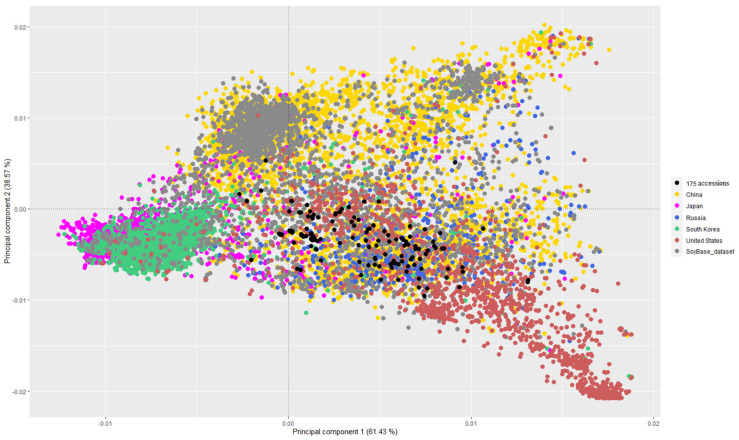
Principal component analysis showing similarities and differences between 175 breeding lines, varieties cultivated in Russia and varieties from other countries (described in the legend on the right) (for detail see Supplementary Table S1).

**Figure 6:**
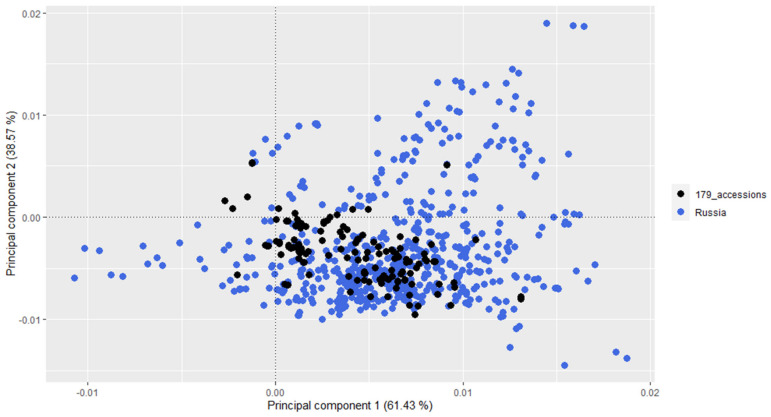
Principal component analysis showing similarities and differences between 175 Russian soybean varieties and Russian varieties from SoyBase.

We merged our dataset with the publicly available *G. max* dataset SoyBase. Principal component analysis of the merged dataset is shown in Figure 5. The 175 soybean accessions cultivated in West Siberia as well as those cultivated in other regions of Russia, represented by black dots, were located closer to each other in the PCA plot. This observation suggests that these accessions are possibly genetically similar. Also, the analyzed 175 accessions are similar to other varieties from Russia in SoyBase (Figure 6).

## 4. Discussion

In this study, we have performed a comprehensive population genetics examination of breeding lines and varieties cultivated in West Siberia and other regions of Russia and compared them with the genotypes from other regions. The overall results of admixture analysis are in correspondence with the result from a similar analysis of Russian varieties in [33]. Four clusters detected in our admixture analysis most probably correspond to four main genetic ancestral populations [33]. Samples in each cluster precisely coincide with the PCA results, where four clusters were also observed (Figure 2): two clusters on the left of the figure are clearly separated while two clusters on the right might be separated using admixture analysis results. Hence, samples above zero value on the *Y* axis can be attributed to one cluster and above that—to another.

Comparison between breeding lines and varieties cultivated in Russia, and varieties from other countries (Figure 5), shows that studied accessions are genetically close to varieties from China, which was already shown in other studies (e.g., [34]), Japan, and the USA, and they differ from varieties from South Korea. Japan and Korea have a long history of soybean cultivation and possess their own rich soybean gene pool [35]. Moreover, there is an idea that there had been independent domestication of wild soybeans [36]. According to the samples included in the SoyBase database, South Korea prefers to use its own unique gene pool (Figure 5), while in Japan, the soybean collection expands by attracting varieties from other countries. Also, we observed a smaller diversity within the studied 179 accessions as compared to those from China, Japan, and the USA. This might be due to a small number of accessions.

The first attempts at soybean cultivation in Russia were made in the early 20th century in the Amur region. For a hundred years, almost all the soybean-sown areas have been concentrated in this region, and the most extensive soybean breeding has been carried out there. As in the USA, where the founders of American cultivars originated from China [33], in the Amur region varieties introduced from China also initially acted as the source material for breeding [37]. Many selected varieties with the origin from the Amur region were later used for breeding in other regions of Russia.

Attempts to introduce soybeans in Siberia have been made since the early 1920s [38]. The main trend in soybean breeding for West Siberia was the creation of precocious varieties, allowing them to avoid low temperatures that are detrimental to soybeans in May and September. It can be expected that precocious varieties of the Siberian soybean ecotype, which are adaptive to growing conditions in a sharply continental climate at lower temperatures and with a long light day, should have genomic differences with Far Eastern and European soybean ecotypes. These differences are clearly seen from the PCA (Figure 2, Table S2). At least two clusters (in the left part of the figure) consist mainly of samples from the Western Siberia breeding varieties, while the third and fourth clusters (in the right part of the figure) are enriched with Far Eastern and European varieties, respectively. The isolation of some samples adapted to the conditions of Western Siberia can also be seen in comparison with the varieties from the SoyBase database. For instance, in the square with PCA1 (coordinates from −0.0005 till +0.00447) and PCA2 (coordinates from +0.001 till −0.0042), there are mainly varieties of the West Siberian selection (Novosibirsk, Omsk) (Figure 6).

The first Siberian soybean variety SibNIIK-315 was selected by individual selection of the Swedish sample from the Federal Research Center “N.I. Vavilov All-Russian Institute of Plant Genetic Resources” collection. SibNIIK-315 and its line SibNIIK-315_st_9 were included in this study as a standard variety and line, respectively. Interestingly, these samples were separated into different clusters (Table S2), but their genetic relationship with the samples of Swedish origin has been preserved, since Swedish samples are also present in both of these clusters.

It could be problematic to compare some population genetics parameters that were used to describe soybean varieties cultivated in Russia before because of the different genotyping technology and coverage used (i.e., set of genes, SNP arrays, transcriptomic, or whole-genome sequencing data). Nevertheless, in comparable studies, it was found that in domesticated soybeans, the mean per-site π was 0.189, and in *G. max*, it was equal to 0.23 [35], while in our study it was 0.33, probably due to higher diversity of the dataset used before. In [14], θ for cultivar soybean was 0.21, while for landrace soybean was —0.27, while in our study the same value was 0.17. This difference can be attributed to the fact that in the previous study, WGS data were used, which can lead to higher values of θ. As Tajima’s D value in our dataset is 2.94, it means that the data show deviation from the neutral expectation (D  =  0) and that rare alleles are presented at low frequencies simultaneously with the excess of common variants. Positive Tajima’s D value was also observed in [7,14]. In some research works, this value was shown as negative [11], and various studies illustrated how this value can vary between genes [39]. Also, we observed very low heterozygosity, which agrees with current research [17,40].

There is some discordance with the estimated LD decay and half-life in other studies. For all chromosomes, the LD decay was estimated to be 0.33 Mb and LD half-life as 1.2 Mb. For each chromosome separately, LD half-life varies from 0.49 for chromosome 3 to 10.56 for chromosome 14 (Supplementary Figures S5–S24). Some studies using WGS data indicated that LD half-life is 420 Kb [34], while similar long distance LD decay was also observed [40]. It raises a question of whether these differences are due to differing datasets and applied filter criteria or due to genetically mediated differences between studied populations.

This study has several limitations. First, the dataset does not include wild accessions that could be of special importance as a potential source of genetic diversity for the selection. Second, the analysis was performed on genotypes obtained with SNP-array technology. Obviously, results obtained with WGS could potentially be more robust, descriptive, and comparable with other studies. Future studies will remove these limitations and widen our knowledge about soybean breeding lines and varieties cultivated in Russia.

Overall, in this study, we present the first population structure and genetic diversity analysis of the 175 breeding lines and varieties cultivated in Russia, among them 103 accessions cultivated in West Siberia, and compare them with the cultivated soybean varieties from other locations. These results provide information about their genetic similarity and origin that can be further used for selection and in consequent genetic analyses like QTL mapping.

## Figures and Tables

**Figure 4:**
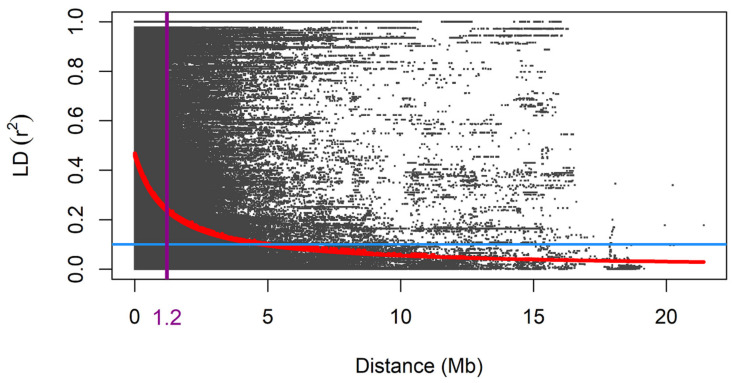
LD (r^2^) decay in 175 studied soybean accessions. LD half-life is shown with violet line and equals to 1.2 Mb. LD (r^2^) value of 0.1 is highlighted by blue line, and red line shows nonlinear regression of r^2^ on weighted distance.

## Data Availability

The data presented in this study are available on request from the corresponding author.

## Supplementary Materials

The following supporting information can be downloaded at https://www.mdpi.com/article/10.3390/plants12193490/s1, Figure S1: Distribution of 29 724 SNPs among chromosomes in 175 soybean accessions; Figure S2: Allele frequency distribution presented for 175 soybean accessions; Figure S3: Percent of the genetic variation explained by the first 10 principal components; Figure S4: Relationship between K parameter and cross-validation error; Figure S5: LD (r2) decay in 1 chromosome of 175 soybean accessions. LD half-life is shown with green line, LD (r2) value of 0.1 is highlighted by blue line, red line shows nonlinear regression of r2 on weighted distance; Figure S6: LD (r2) decay in 2 chromosome of 175 soybean accessions. LD half-life is shown with green line, LD (r2) value of 0.1 is highlighted by blue line, red line shows nonlinear regression of r2 on weighted distance; Figure S7: LD (r2) decay in 3 chromosome of 175 soybean accessions. LD half-life is shown with green line, LD (r2) value of 0.1 is highlighted by blue line, red line shows nonlinear regression of r2 on weighted distance; Figure S8: LD (r2) decay in 4 chromosome of 175 soybean accessions. LD half-life is shown with green line, LD (r2) value of 0.1 is highlighted by blue line, red line shows nonlinear regression of r2 on weighted distance; Figure S9: LD (r2) decay in 1 chromosome of 175 soybean accessions. LD half-life is shown with green line, LD (r2) value of 5 is highlighted by blue line, red line shows nonlinear regression of r2 on weighted distance; Figure S10: LD (r2) decay in 6 chromosome of 175 soybean accessions. LD half-life is shown with green line, LD (r2) value of 0.1 is highlighted by blue line, red line shows nonlinear regression of r2 on weighted distance; Figure S11: LD (r2) decay in 7 chromosome of 175 soybean accessions. LD half-life is shown with green line, LD (r2) value of 0.1 is highlighted by blue line, red line shows nonlinear regression of r2 on weighted distance; Figure S12: LD (r2) decay in 8 chromosome of 175 soybean accessions. LD half-life is shown with green line, LD (r2) value of 0.1 is highlighted by blue line, red line shows nonlinear regression of r2 on weighted distance; Figure S13: LD (r2) decay in 9 chromosome of 175 soybean accessions. LD half-life is shown with green line, LD (r2) value of 0.1 is highlighted by blue line, red line shows nonlinear regression of r2 on weighted distance; Figure S14: LD (r2) decay in 10 chromosome of 175 soybean accessions. LD half-life is shown with green line, LD (r2) value of 0.1 is highlighted by blue line, red line shows nonlinear regression of r2 on weighted distance; Figure S15: LD (r2) decay in 11 chromosome of 175 soybean accessions. LD half-life is shown with green line, LD (r2) value of 0.1 is highlighted by blue line, red line shows nonlinear regression of r2 on weighted distance; Figure S16: LD (r2) decay in 12 chromosome of 175 soybean accessions. LD half-life is shown with green line, LD (r2) value of 0.1 is highlighted by blue line, red line shows nonlinear regression of r2 on weighted distance; Figure S17: LD (r2) decay in 13 chromosome of 175 soybean accessions. LD half-life is shown with green line, LD (r2) value of 0.1 is highlighted by blue line, red line shows nonlinear regression of r2 on weighted distance; Figure S18: LD (r2) decay in 14 chromosome of 175 soybean accessions. LD half-life is shown with green line, LD (r2) value of 0.1 is highlighted by blue line, red line shows nonlinear regression of r2 on weighted distance; Figure S19: LD (r2) decay in 15 chromosome of 175 soybean accessions. LD half-life is shown with green line, LD (r2) value of 0.1 is highlighted by blue line, red line shows nonlinear regression of r2 on weighted distance; Figure S20: LD (r2) decay in 16 chromosome of 175 soybean accessions. LD half-life is shown with green line, LD (r2) value of 0.1 is highlighted by blue line, red line shows nonlinear regression of r2 on weighted distance; Figure S21: LD (r2) decay in 17 chromosome of 175 soybean accessions. LD half-life is shown with green line, LD (r2) value of 0.1 is highlighted by blue line, red line shows nonlinear regression of r2 on weighted distance; Figure S22: LD (r2) decay in 18 chromosome of 175 soybean accessions. LD half-life is shown with green line, LD (r2) value of 0.1 is highlighted by blue line, red line shows nonlinear regression of r2 on weighted distance; Figure S23: LD (r2) decay in 19 chromosome of 175 soybean accessions. LD half-life is shown with green line, LD (r2) value of 0.1 is highlighted by blue line, red line shows nonlinear regression of r2 on weighted distance; Figure S24: LD (r2) decay in 20 chromosome of 175 soybean accessions. LD half-life is shown with green line, LD (r2) value of 0.1 is highlighted by blue line, red line shows nonlinear regression of r2 on weighted distance; Table S1: Information about the soybean accessions; Table S2: Distrubution of the analyzed accessions across clusters of Admixture analysis. For each cluster are shown accession name (first column) and origin (second column), respectively. Color for each cluster corresponds to color from the Figure 3.
