# Supplementary material for: Population Structure and Genetic Diversity of the 175 Soybean Breeding Lines and Varieties Cultivated in West Siberia and Other Regions of Russia
Source: Plants (Basel). 2023 Oct 6;12(19):3490. doi: 10.3390/plants12193490 (PMC10575349; doi:10.3390/plants12193490)
Supplement: Supplementary file 1 [file plants-12-03490-s001.zip › Suppl_Figures_Soybean.pdf]

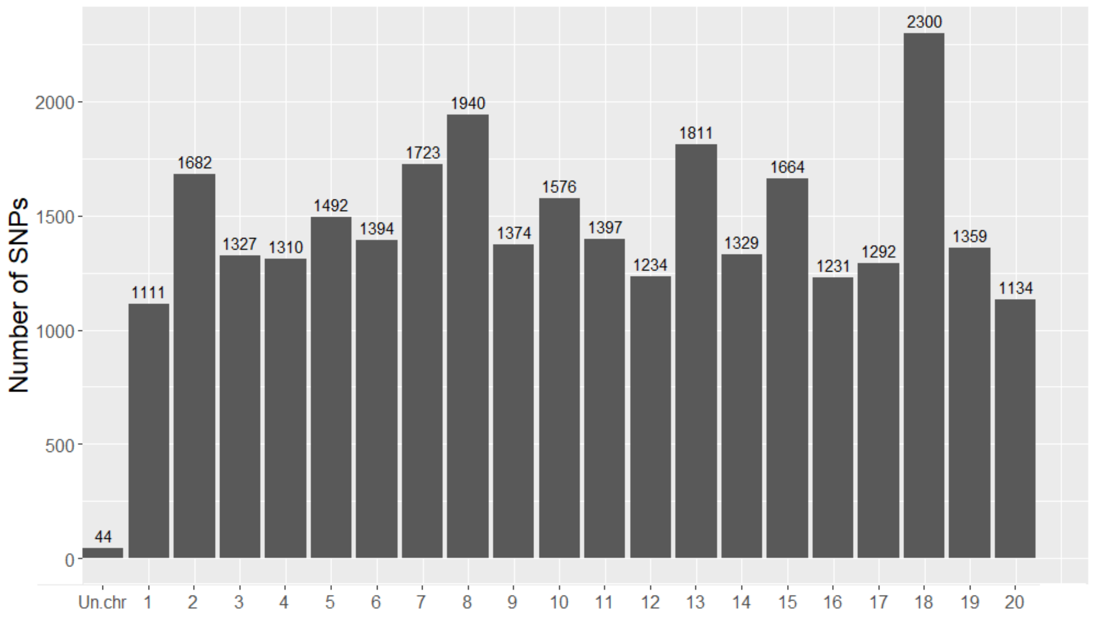

Supplementary Figure S1. Distribution of 29 724 SNPs among chromosomes in 175 soybean accessions.

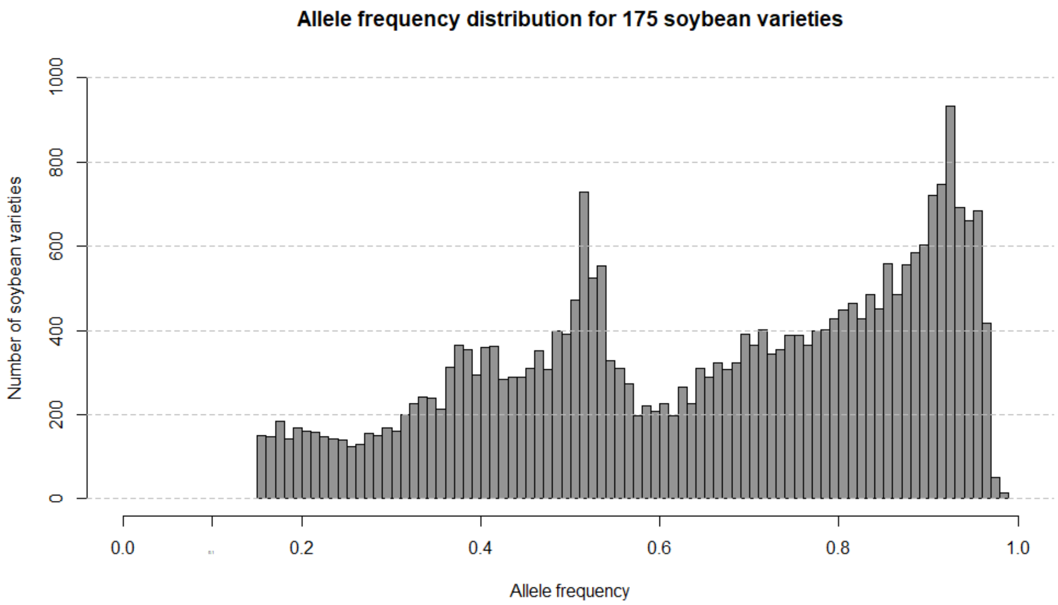

Supplementary Figure S2. Allele frequency distribution presented for 175 soybean accessions.

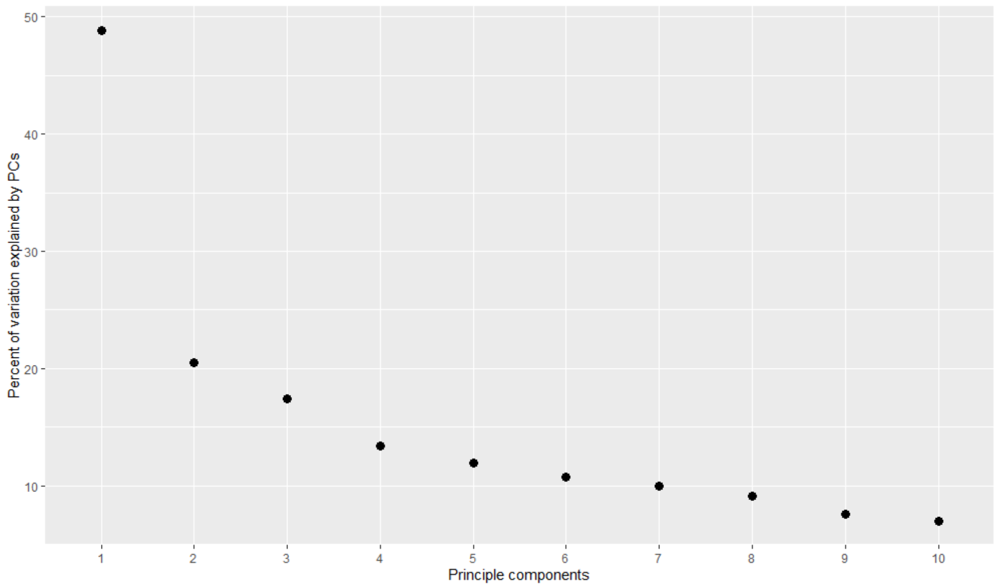

Supplementary Figure S3. Percent of the genetic variation explained by the first 10 principal components.

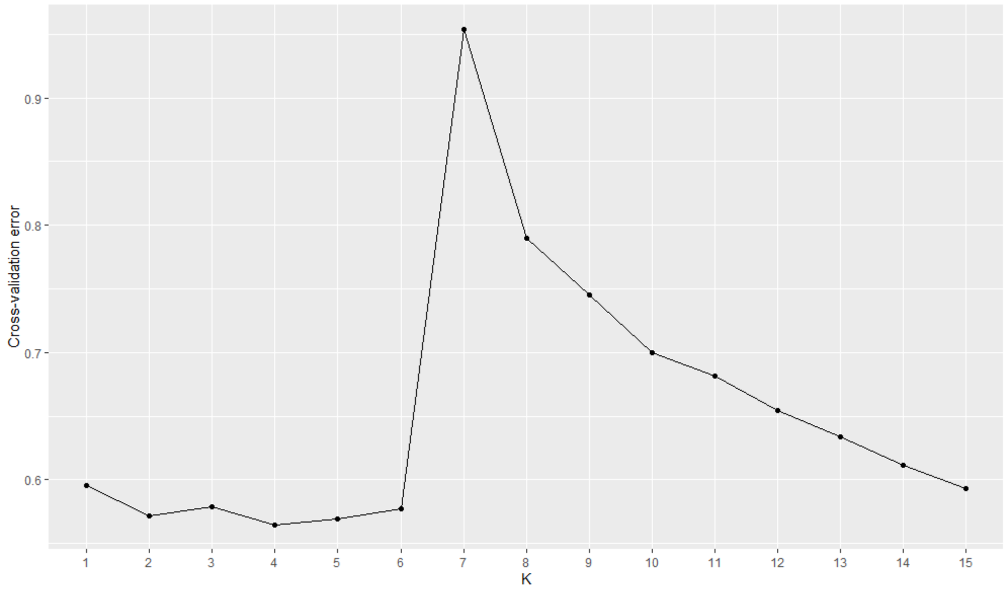

Supplementary Figure S4. Relationship between K parameter and cross-validation error.

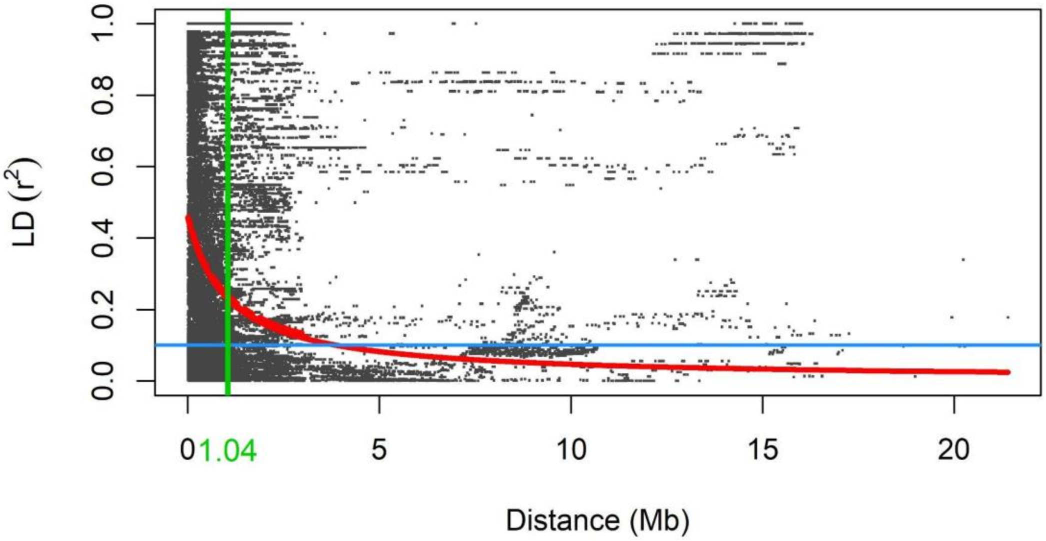

Supplementary Figure S5. LD (r^2) decay in 1 chromosome of 175 soybean accessions. LD half-life is shown with green line, LD (r^2) value of 0.1 is highlighted by blue line, red line shows nonlinear regression of r^2 on weighted distance.

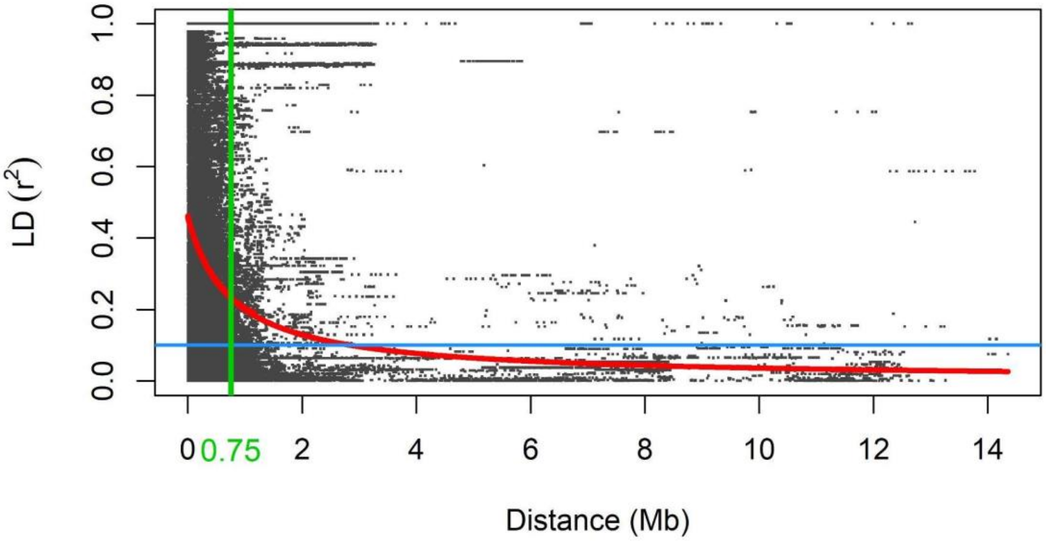

Supplementary Figure S6. LD (r^2) decay in 2 chromosome of 175 soybean accessions. LD half-life is shown with green line, LD (r^2) value of 0.1 is highlighted by blue line, red line shows nonlinear regression of r^2 on weighted distance.

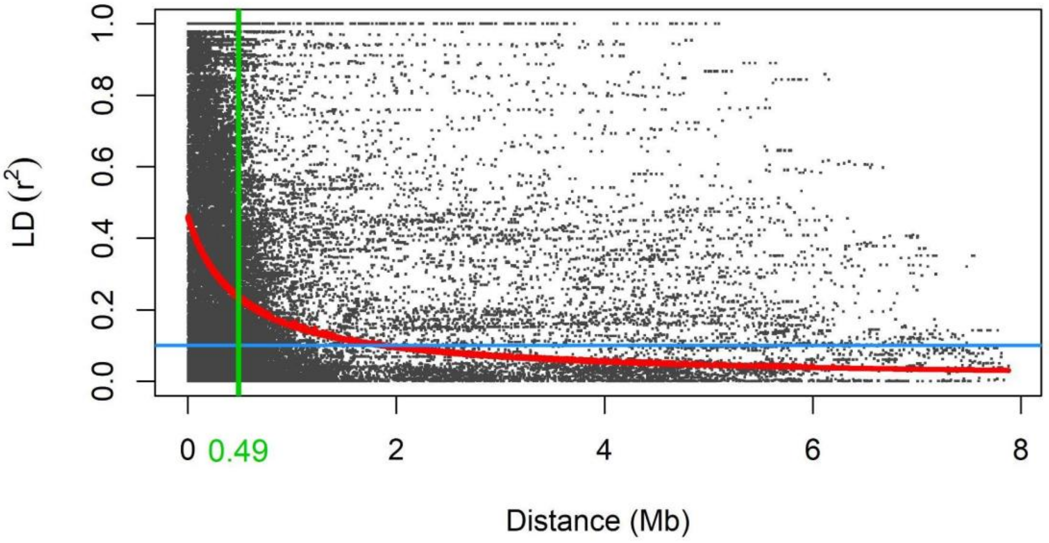

Supplementary Figure S7. LD (r^2) decay in 3 chromosome of 175 soybean accessions. LD half-life is shown with green line, LD (r^2) value of 0.1 is highlighted by blue line, red line shows nonlinear regression of r^2 on weighted distance.

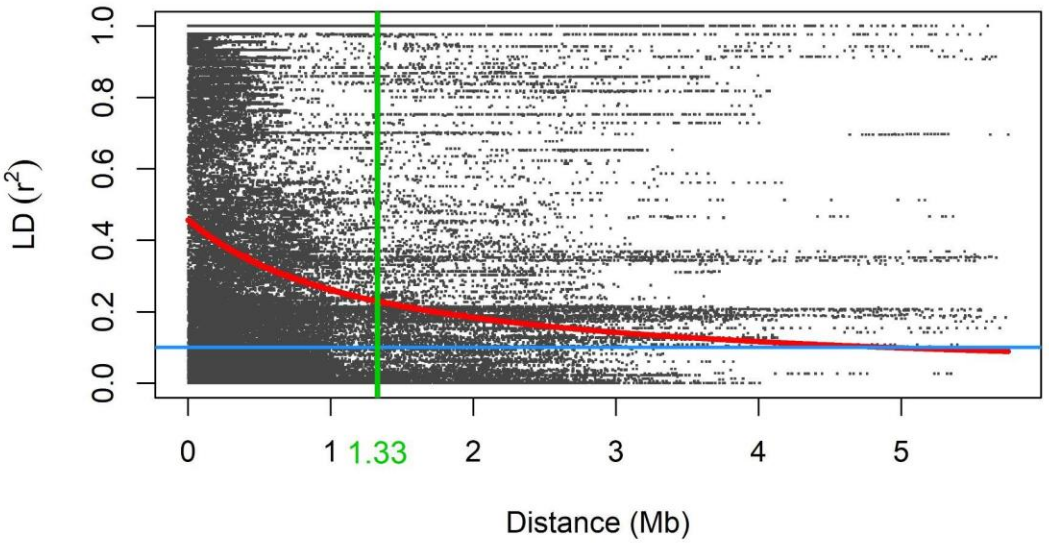

Supplementary Figure S8. LD (r^2) decay in 4 chromosome of 175 soybean accessions. LD half-life is shown with green line, LD (r^2) value of 0.1 is highlighted by blue line, red line shows nonlinear regression of r^2 on weighted distance.

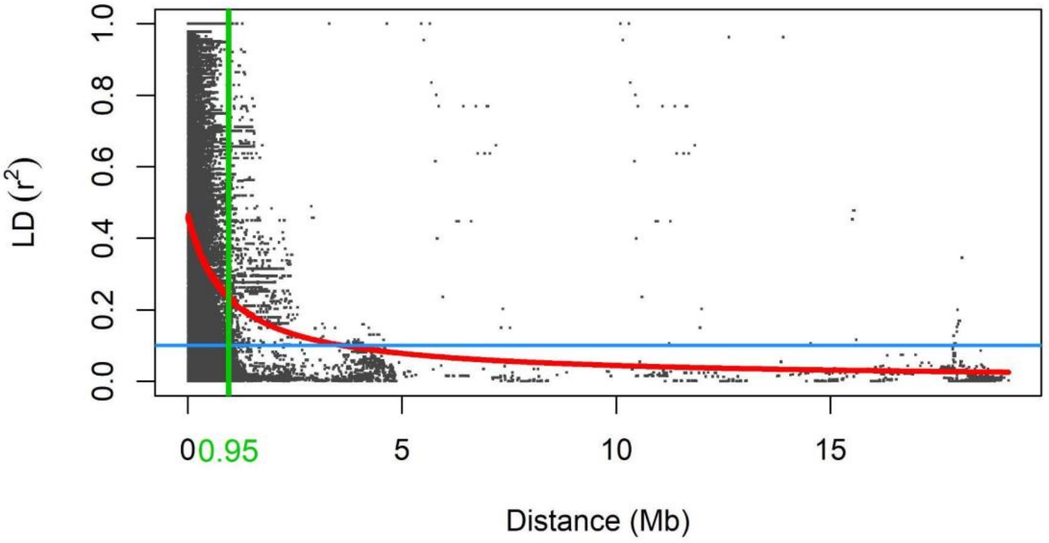

Supplementary Figure S9. LD (r^2) decay in 5 chromosome of 175 soybean accessions. LD half-life is shown with green line, LD (r^2) value of 0.1 is highlighted by blue line, red line shows nonlinear regression of r^2 on weighted distance.

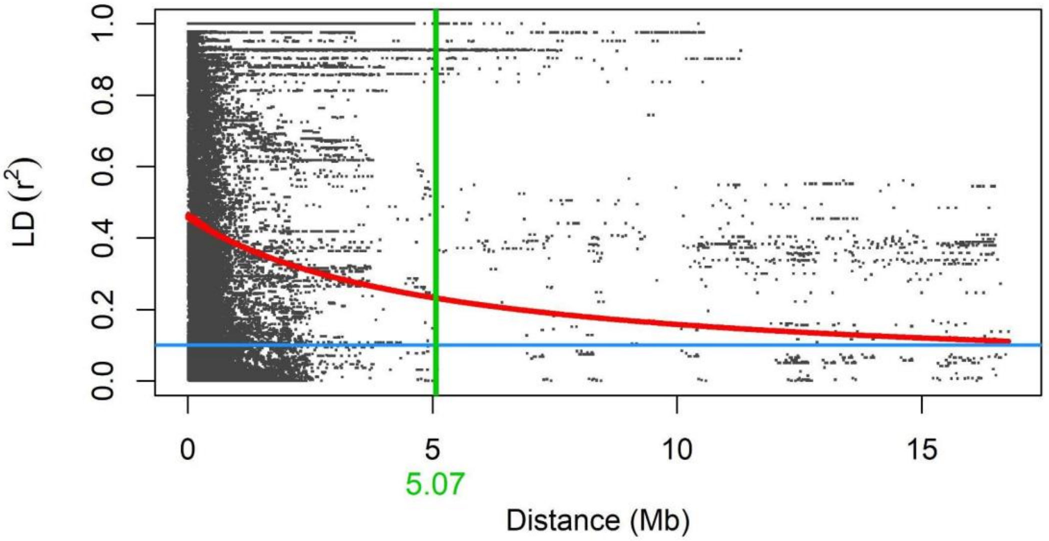

Supplementary Figure S10. LD (r^2) decay in 6 chromosome of 175 soybean accessions. LD half-life is shown with green line, LD (r^2) value of 0.1 is highlighted by blue line, red line shows nonlinear regression of r^2 on weighted distance.

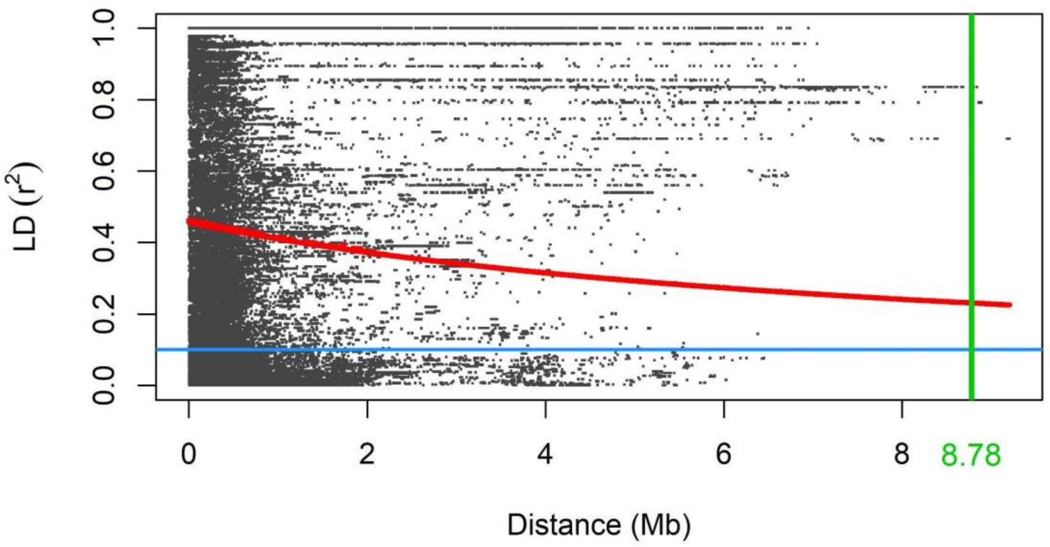

Supplementary Figure S11. LD (r^2) decay in 7 chromosome of 175 soybean accessions. LD half-life is shown with green line, LD (r^2) value of 0.1 is highlighted by blue line, red line shows nonlinear regression of r^2 on weighted distance.

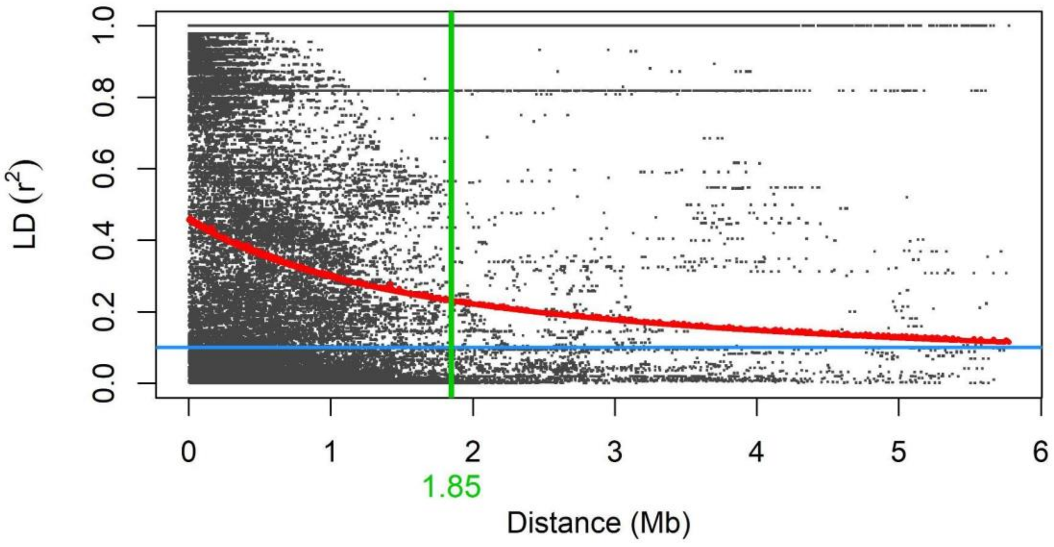

Supplementary Figure S12. LD (r^2) decay in 8 chromosome of 175 soybean accessions. LD half-life is shown with green line, LD (r^2) value of 0.1 is highlighted by blue line, red line shows nonlinear regression of r^2 on weighted distance.

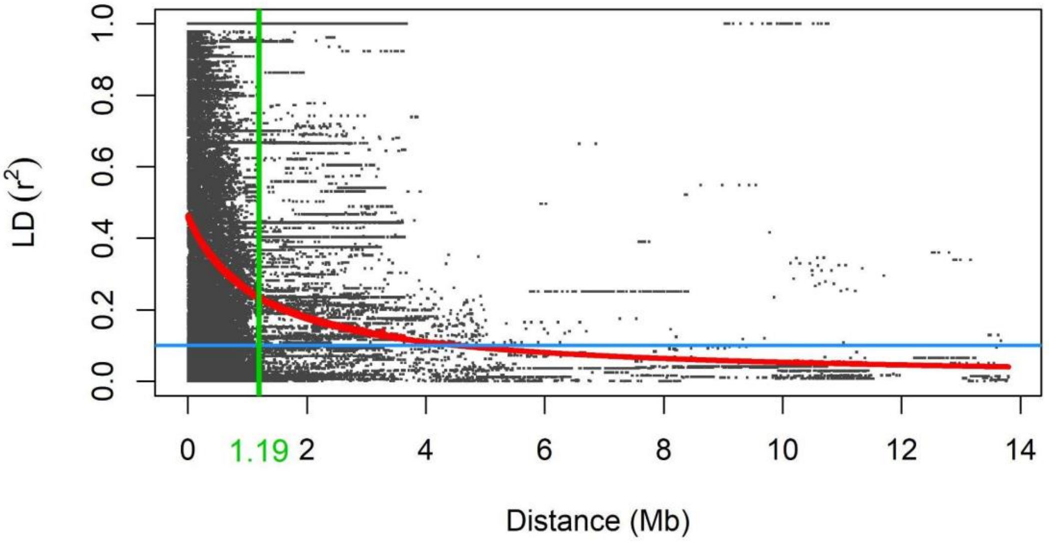

Supplementary Figure S13. LD (r^2) decay in 9 chromosome of 175 soybean accessions. LD half-life is shown with green line, LD (r^2) value of 0.1 is highlighted by blue line, red line shows nonlinear regression of r^2 on weighted distance.

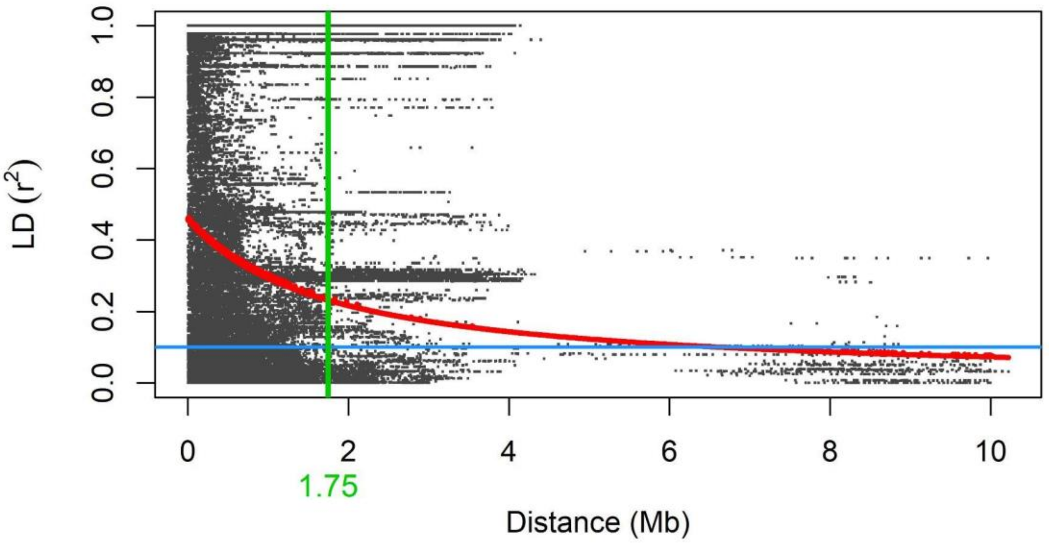

Supplementary Figure S14. LD (r^2) decay in 10 chromosome of 175 soybean accessions. LD half-life is shown with green line, LD (r^2) value of 0.1 is highlighted by blue line, red line shows nonlinear regression of r^2 on weighted distance.

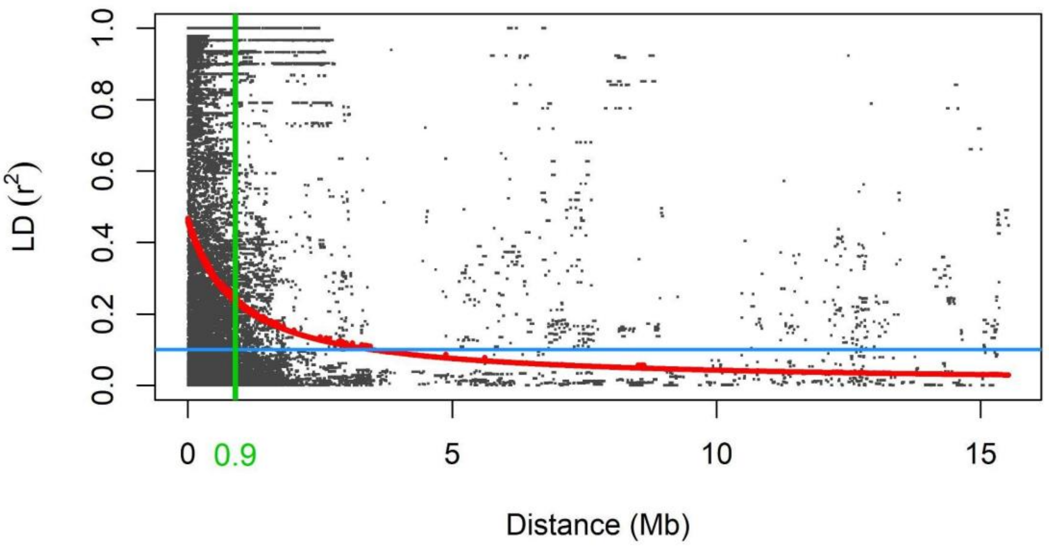

Supplementary Figure S15. LD (r^2) decay in 11 chromosome of 175 soybean accessions. LD half-life is shown with green line, LD (r^2) value of 0.1 is highlighted by blue line, red line shows nonlinear regression of r^2 on weighted distance.

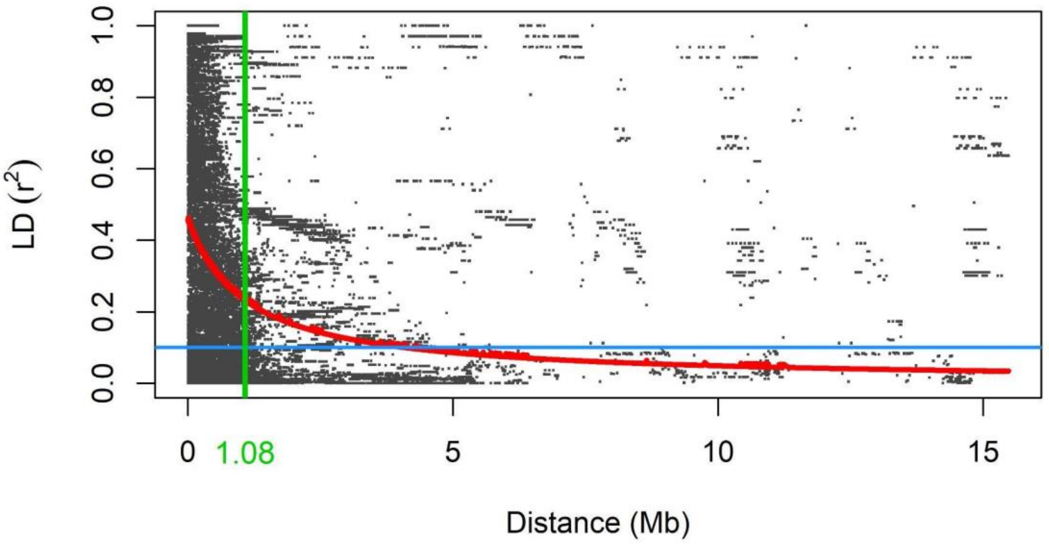

Supplementary Figure S16. LD (r^2) decay in 12 chromosome of 175 soybean accessions. LD half-life is shown with green line, LD (r^2) value of 0.1 is highlighted by blue line, red line shows nonlinear regression of r^2 on weighted distance.

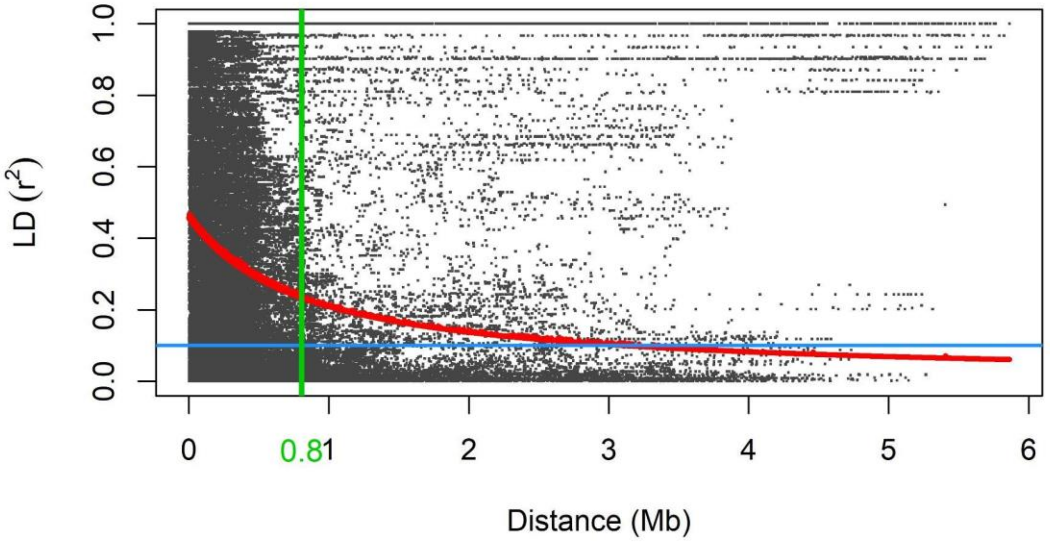

Supplementary Figure S17. LD (r^2) decay in 13 chromosome of 175 soybean accessions. LD half-life is shown with green line, LD (r^2) value of 0.1 is highlighted by blue line, red line shows nonlinear regression of r^2 on weighted distance.

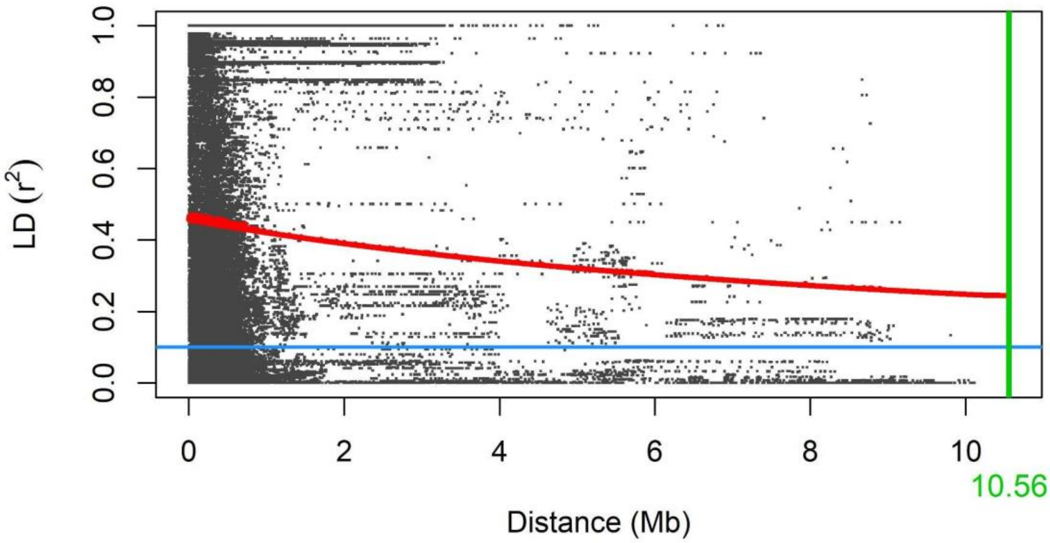

Supplementary Figure S18. LD (r^2) decay in 14 chromosome of 175 soybean accessions. LD half-life is shown with green line, LD (r^2) value of 0.1 is highlighted by blue line, red line shows nonlinear regression of r^2 on weighted distance.

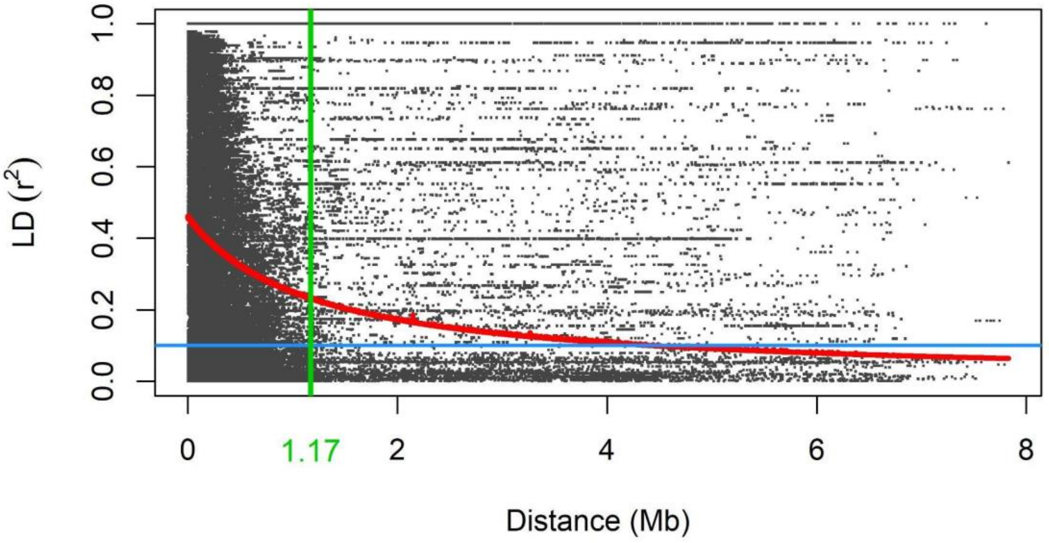

Supplementary Figure S19. LD (r^2) decay in 15 chromosome of 175 soybean accessions. LD half-life is shown with green line, LD (r^2) value of 0.1 is highlighted by blue line, red line shows nonlinear regression of r^2 on weighted distance.

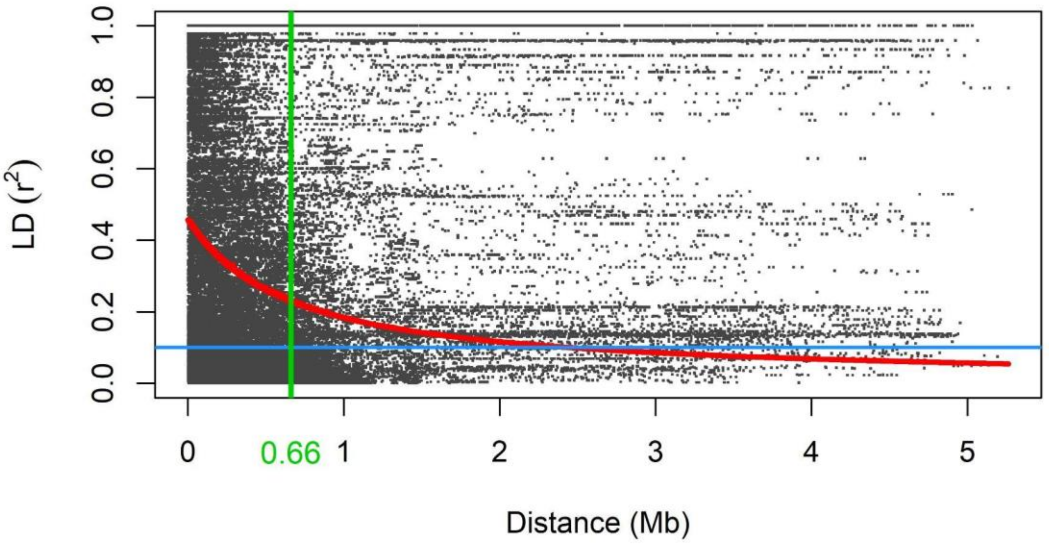

Supplementary Figure S20. LD (r^2) decay in 16 chromosome of 175 soybean accessions. LD half-life is shown with green line, LD (r^2) value of 0.1 is highlighted by blue line, red line shows nonlinear regression of r^2 on weighted distance.

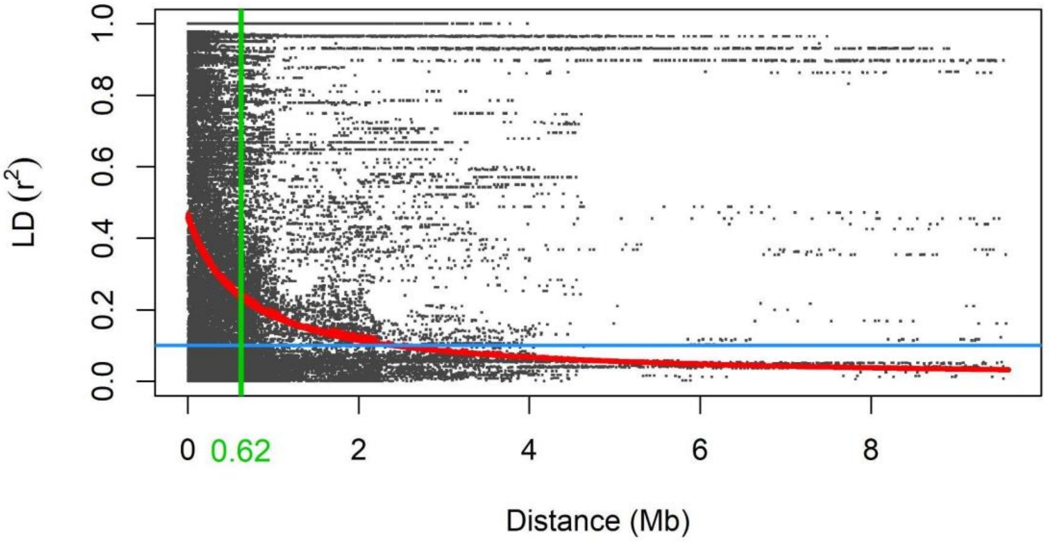

Supplementary Figure S21. LD (r^2) decay in 17 chromosome of 175 soybean accessions. LD half-life is shown with green line, LD (r^2) value of 0.1 is highlighted by blue line, red line shows nonlinear regression of r^2 on weighted distance.

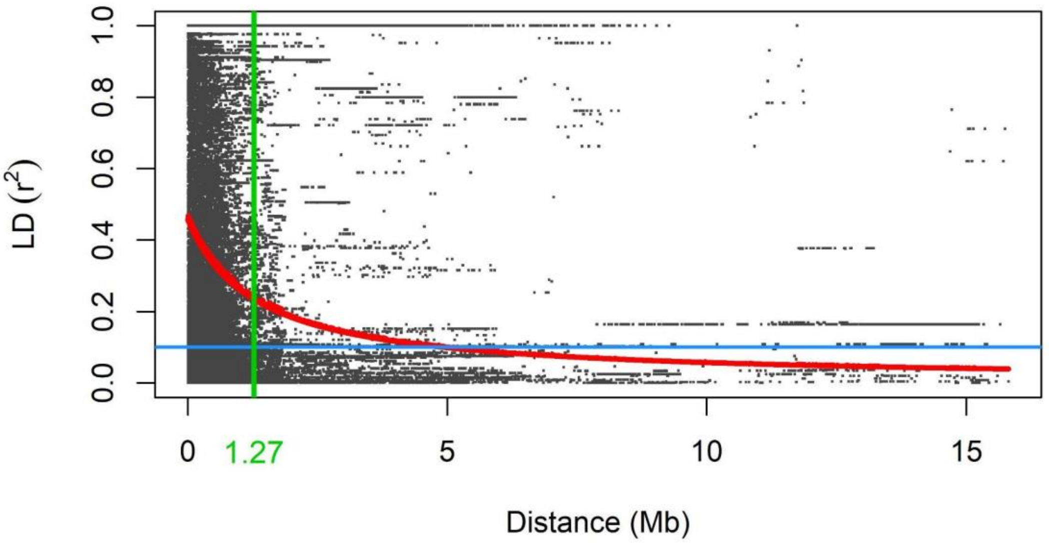

Supplementary Figure S22. LD (r^2) decay in 18 chromosome of 175 soybean accessions. LD half-life is shown with green line, LD (r^2) value of 0.1 is highlighted by blue line, red line shows nonlinear regression of r^2 on weighted distance.

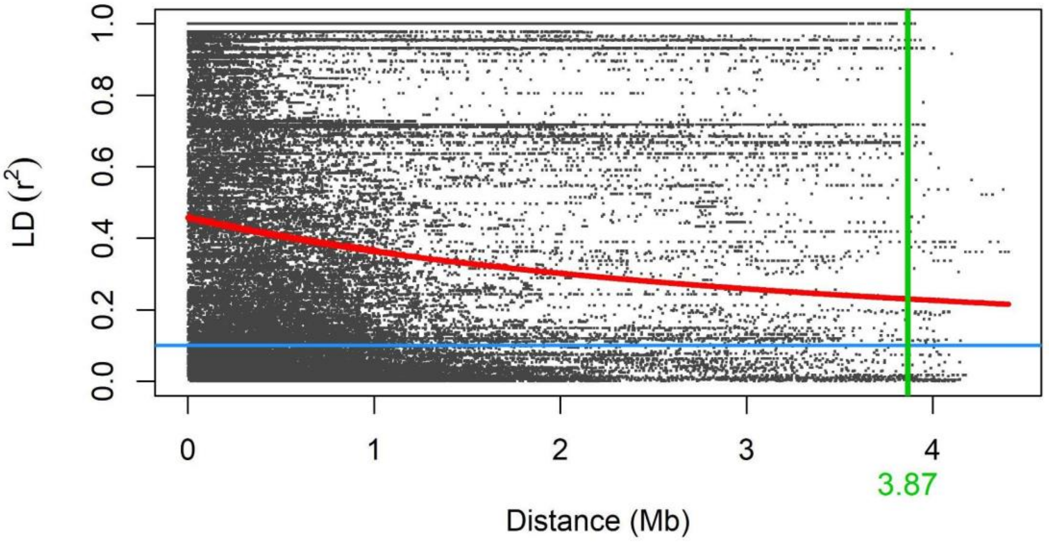

Supplementary Figure S23. LD (r^2) decay in 19 chromosome of 175 soybean accessions. LD half-life is shown with green line, LD (r^2) value of 0.1 is highlighted by blue line, red line shows nonlinear regression of r^2 on weighted distance.

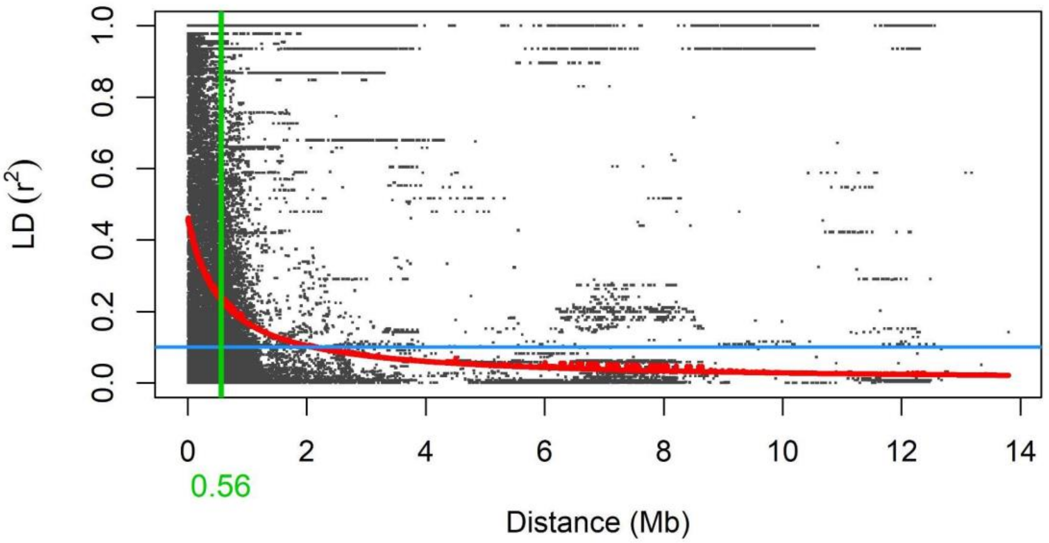

Supplementary Figure S24. LD (r^2) decay in 20 chromosome of 175 soybean accessions. LD half-life is shown with green line, LD (r^2) value of 0.1 is highlighted by blue line, red line shows nonlinear regression of r^2 on weighted distance.
